# Second-Trimester Uterine Rupture Following Misoprostol-Induced Termination in a Woman With a Previous Cesarean Section: A Case Report

**DOI:** 10.7759/cureus.94191

**Published:** 2025-10-09

**Authors:** Ibrahim Kale, Mehtap Günes, Ipek Pehlivan, Melda Kuyucu

**Affiliations:** 1 Obstetrics and Gynecology, Umraniye Training and Research Hospital, Istanbul, TUR; 2 Obstetrics and Gynecology, Faculty of Medicine, Bezmialem Vakif University, Istanbul, TUR

**Keywords:** medical termination of pregnancy, misoprostol, obstetric emergency, second-trimester abortion, uterine rupture

## Abstract

Misoprostol, a prostaglandin E1 analogue, is widely used for medical termination of pregnancy due to its uterotonic properties. Although generally considered safe, uterine rupture is a rare but potentially life-threatening complication, particularly in women with a history of cesarean delivery. We report a case of a 23-year-old gravida 3, para 1 woman with one previous cesarean section who underwent second-trimester medical termination with the International Federation of Gynecology and Obstetrics (FIGO)-recommended misoprostol dosing. After the ninth dose, she developed sudden abdominal pain and syncope-like symptoms. Ultrasonography revealed uterine rupture with the fetus and placenta located in the abdominal cavity. Emergency laparotomy was performed, and the rupture along the previous cesarean scar was repaired. Her postoperative course was uneventful, and she was discharged on day 3. This case underscores the importance of early recognition of uterine rupture during misoprostol-induced second-trimester abortion and highlights the need for prompt surgical preparedness in women with prior uterine surgery.

## Introduction

Misoprostol, a prostaglandin E1 analogue, was initially approved for the prevention of gastric ulcers [1]. However, it was later discovered to possess uterotonic properties and has since been employed off-label in various obstetric settings, including the induction of abortion, cervical ripening, labor induction, and the treatment of postpartum hemorrhage secondary to uterine atony [2]. Its low cost, lack of requirement for cold-chain storage, and ease of administration via oral, vaginal, or rectal routes have made misoprostol one of the most commonly used drugs in many obstetric care settings. Therefore, in 2017, the International Federation of Gynecology and Obstetrics (FIGO) published a statement providing a series of recommendations regarding the use of misoprostol alone in gynecology and obstetrics [3]. This guideline was subsequently revised in 2023 [4].

Complete uterine rupture during pregnancy, although rare, is a life-threatening obstetric emergency associated with maternal and fetal morbidity and mortality [5]. Uterine scars, particularly those resulting from incisions made during cesarean deliveries or myomectomy, are the most prominent risk factors; however, uterine rupture can also occur in women with an unscarred uterus [6,7]. Uterine rupture can occur in a scarred uterus, during a trial of labor, or following medical abortion with pharmacological agents. In women with a previous cesarean delivery, the rate of uterine rupture during a trial of labor has been reported to be approximately 1%, whereas in women with multiple prior cesarean deliveries, this rate has been reported to increase to 3.9% [8]. On the other hand, in women with a previous cesarean delivery who underwent second-trimester abortion using misoprostol, the risk of uterine rupture has been reported to be less than 0.3% [9].

Here, we report a case of a patient who received misoprostol for second-trimester medical termination in accordance with FIGO recommendations and subsequently experienced uterine rupture.

## Case presentation

A 23-year-old gravida 3, para 1 woman with one previous cesarean section presented to the Obstetrics and Gynecology outpatient clinic at Umraniye Training and Research Hospital in Istanbul, Turkey, for routine antenatal follow-up. The patient initially underwent first-trimester screening, which revealed a low risk for trisomies 21, 13, and 18. The patient had undergone a cesarean delivery 1.5 years earlier. Her first child was diagnosed with pontocerebellar hypoplasia type 10, which prompted the decision to perform chorionic villus sampling (CVS) at 13 weeks and one day of the current pregnancy. Genetic analysis of the CVS sample revealed a homozygous CLP1 (NM_006831.3): c.419G>A (p.Arg140His) rs587777616 variant. At 19 weeks of gestation, a detailed fetal anomaly screening ultrasound was performed, yielding normal findings consistent with a 19-week gestation. During a multidisciplinary perinatology council meeting at our hospital, termination of pregnancy was offered based on the genetic findings. Upon the patient’s and her husband’s decision to proceed with termination, informed consent was obtained, and she was hospitalized for medical termination at 19 weeks and three days of gestation, as calculated from her last menstrual period.

According to the 2023 FIGO recommendations on medical termination of pregnancy, the patient was started on a misoprostol regimen consisting of 200 μg orally and 200 μg vaginally (total 400 μg) administered every three hours [4]. During the course of misoprostol administration, the patient reported no complaints other than mild inguinal pain and exhibited no vaginal bleeding. This discomfort was attributed to cervical effacement and dilatation secondary to the effect of misoprostol. The patient received nine consecutive doses without discontinuation. However, after the ninth dose of misoprostol, the patient’s clinical condition deteriorated abruptly. She developed nausea and vomiting, accompanied by severe abdominal pain, most pronounced in the suprapubic region, and a sensation of impending syncope. On manual examination, diffuse abdominal tenderness was noted. Bedside assessment of vital signs revealed hypotension (80/40 mmHg) and tachycardia (128 beats/min). Vaginal examination showed no cervical effacement but approximately 2 cm of cervical dilatation, along with the onset of vaginal bleeding. Ultrasonographic evaluation revealed uterine rupture, with the fetus and placenta visualized within the abdominal cavity. The patient and her relatives were informed about her condition, and after obtaining the necessary informed consent, she was taken for emergency laparotomy under general anesthesia. The abdomen was entered through an incision along the previous cesarean scar, and the abdominal layers were opened in the standard manner. Intra-abdominal examination revealed active bleeding, along with the fetus and placenta. The fetus and placenta were removed, and the uterus was exteriorized. Inspection revealed a rupture along the previous cesarean scar line (Video 1). The rupture site was repaired in the standard procedure. After thorough peritoneal lavage, the abdominal cavity was closed anatomically and in accordance with surgical principles. Intraoperatively, the patient received two units of erythrocyte suspension and two units of fresh frozen plasma. The postoperative course was uneventful, and she was discharged on postoperative day 3.

**Video 1:** Laparotomy showing uterine rupture at the previous cesarean scar during the second trimester, following a misoprostol-induced termination.

## Discussion

The use of misoprostol for pregnancy termination was first reported in the literature in 1994 [10]. Since that time, misoprostol has been widely regarded as a safe and effective agent for termination of pregnancy in both the first and second trimesters. Over the past decades, the rate of cesarean deliveries has increased significantly worldwide due to various factors [11]. Considering that the most important risk factor for uterine rupture is a surgical incision in the uterus from a previous cesarean delivery, it is not surprising that the incidence of uterine rupture has increased in cases of second-trimester pregnancy termination with misoprostol. According to the literature, the majority of uterine rupture cases reported during second-trimester termination with misoprostol have been managed surgically through uterine repair [12,13]. However, cases requiring hysterectomy have also been reported [14,15]. In the case presented here, the uterine rupture was identified early and managed successfully through surgical repair of the uterus following emergency intervention.

In a 2025 review including 164 studies, the incidence of uterine rupture associated with misoprostol was reported as 0% in first-trimester pregnancy terminations. In second-trimester terminations, the incidence was reported as 0.5% in women with a history of one cesarean delivery and 2.2% in those with two or more prior cesarean deliveries. Accordingly, the authors stated that medical abortion is generally safe in both the first and second trimesters for women with a previous cesarean section; however, risks include uterine rupture, the need for surgical intervention, and hemorrhage due to undiagnosed placenta accreta. They emphasized the need for further research on abortion management in women with more than three prior cesarean deliveries or those with an abnormally invasive placenta [16].

## Conclusions

Misoprostol is a safe and effective agent for termination of both first- and second-trimester pregnancies. However, even when administered at FIGO-recommended doses, the risk of uterine rupture should be carefully considered, particularly in patients with a history of multiple cesarean deliveries or other uterine surgeries. In such cases, obstetric centers must be prepared for prompt surgical intervention to manage potential complications. Early recognition and rapid response are essential to ensure optimal maternal outcomes.
